# EFFICACY OF COMBINED THERAPEUTIC EXERCISE AND PSYCHOLOGICAL INTERVENTIONS ON DISABILITY OUTCOMES: A SCOPING REVIEW

**DOI:** 10.1093/geroni/igad104.2456

**Published:** 2023-12-21

**Authors:** Grace Rose, Pietra Bruni, Mariana Wingood, Selmi Kallmi, Elizabeth Finer, Patricia Bamonti

**Affiliations:** VA Boston Healthcare System, Boston, Massachusetts, United States; VA Boston, Boston, Massachusetts, United States; Wake Forest University, Winston-Salem, North Carolina, United States; Hines VA, Hines, Illinois, United States; Hofstra University, Hempstead, New York, United States; VA Boston Healthcare System, Boston, Massachusetts, United States

## Abstract

The goal of therapeutic exercise (i.e., physical activity/exercise programs, physical therapy, rehabilitation), restoring and maintaining function in older adults (≥60 years), is hindered by personal factors (e.g., low self-efficacy) impacting exercise engagement. Combining therapeutic exercise with psychological interventions is a promising solution for maximizing treatment benefits. The purpose of this scoping review is to systematically synthesize the knowledge of randomized controlled trials (RCTs) testing combined therapeutic exercise and psychological interventions targeting disability outcomes in older adults. A comprehensive review of the literature, searching four databases, was performed following PRISMA-ScR guidelines. Articles (n=4,995) underwent iterative screening, resulting in 31 RCTs. The World Health Organization’s International Classification of Functioning, Disability, and Health (ICF) model was utilized as a conceptual framework. Studies assessed at least one major domain of the ICF model: Body Function and Structure was an outcome in 67.74% of RCTs and both Activity and Participation were an outcome in 77.42% of RCTs. Personal factors were assessed in 64.52% of RCTs. Psychological interventions utilized were cognitive-behavioral (32.26%), motivational interviewing (29.03%), meditation (12.90%), or other (32.26%). Combined interventions compared to either therapeutic exercise alone (25.81%) or control (74.19%) demonstrated greater efficacy on at least one major ICF outcome in 77.42% of RCTs. This review indicates that incorporating psychological interventions within therapeutic exercise can maximize treatment benefits in older adults across diagnoses and setting. Future research is needed to better understand which psychological interventions demonstrate greatest efficacy when paired with therapeutic exercise within certain patient subgroups, in order to guide best practice guidelines.

